# Attitudes to and Understanding of Risk of Acquisition of HIV Over Time: Design and Methods for an Internet-based Prospective Cohort Study Among UK Men Who Have Sex With Men (the AURAH2 Study)

**DOI:** 10.2196/resprot.5582

**Published:** 2016-06-15

**Authors:** Janey Sewell, Andrew Speakman, Andrew N Phillips, Valentina Cambiano, Fiona C Lampe, Richard Gilson, David Asboe, Nneka Nwokolo, Amanda Clarke, Ali Ogilvy, Simon Collins, Alison J Rodger

**Affiliations:** ^1^ Department of Infection and Population Health UCL London United Kingdom; ^2^ Chelsea and Westminster Hospital NHS Foundation Trust London United Kingdom; ^3^ Brighton and Sussex University Hospitals NHS Trust Brighton United Kingdom; ^4^ HIV i-Base London United Kingdom

**Keywords:** HIV infection, HIV negative, HIV transmission, HIV testing, men who have sex with men, sexual risk behaviour, pre-exposure prophylaxis, recreational drug use, chemsex, HIV self-testing, health and well-being, study design

## Abstract

**Background:**

The annual number of new human immunodeficiency virus (HIV) infections among men who have sex with men (MSM) has risen in the United Kingdom and, of those who are HIV positive, the proportion undiagnosed is high.

**Objective:**

The prospective AURAH2 study aims to assess factors associated with HIV acquisition among MSM in the United Kingdom and to investigate changes over time within individuals in sexual behavior and HIV-testing practices.

**Methods:**

AURAH2 is a prospective study among MSM without diagnosed HIV, aiming to recruit up to 1000 sexually active MSM attending sexual health clinics in London and Brighton in the United Kingdom. Participants complete an initial paper-based questionnaire, followed by online follow-up questionnaires every 4 months collecting sociodemographic, health and behavioral data, including sexual behavior, recreational and other drug use, HIV testing practices, and pre-exposure prophylaxis use, over a planned 3-year period.

**Results:**

The study is ongoing.

**Conclusions:**

The results from AURAH2 study will provide important insight into established and emerging risk behaviors that may be associated with acquisition of HIV in MSM in the United Kingdom, changes over time within individuals in sexual behavior, and information on HIV testing practices. These data will be crucial to inform future HIV prevention strategies.

## Introduction

### Background

In 2014, the number of men who have sex with men (MSM) that were newly diagnosed with human immunodeficiency virus (HIV) continued to rise with 3360 new diagnoses in the United Kingdom [1]. Currently, there are an estimated 43,500 MSM living with HIV, of whom around 16% are undiagnosed [2]. It is thought that MSM unaware of their HIV infection disproportionately contribute to onward transmission (60-82% of new transmissions come from people not diagnosed [3,4]) and that delay in diagnosis and treatment is associated with increased risk to health [5]. HIV prevention approaches have historically focused on condom use, which, if correctly and consistently used, is a reliable and established method to reduce transmission [6]; however, consistent condom use is difficult to achieve [7,8]. There is a clear need for improved HIV prevention and testing strategies targeted at HIV negative MSM to reduce the number of new HIV infections and increase HIV testing rates.

The AURAH study was a cross-sectional questionnaire study that collected data from 2013-2014 in a large sample of HIV-negative patients attending Genito-Urinary Medicine (GUM) clinics in the United Kingdom with a focus on two populations: black Africans and MSM [9]. It used a self-completed questionnaire to assess knowledge of and attitudes to HIV transmission risks and the role of antiretroviral therapy (ART), and to assess the prevalence of medical and psychological symptoms (eg, depression and anxiety), quality of life, lifestyle factors (eg, drug and alcohol use), and possible links to sexual risk behaviors. The AURAH2 study will build on the work of the AURAH study and is the first large prospective observational study of MSM in the United Kingdom. It will provide longitudinal data on HIV transmission risk in a group of HIV negative (at enrollment) MSM using online questionnaires for data collection over a 3-year period. It will collect baseline socioeconomic, health and lifestyle information (including recreational drug use and chemsex) with longitudinal information on sexual activity, HIV testing, sexual behavior, and occurrence of new HIV infections among UK MSM.

Understanding attitudes of HIV negative or undiagnosed MSM towards condomless sex with individuals of unknown HIV status, and examining risk behavior in the context of psychological or general health status, history of sexually transmitted infection (STI), alcohol and drug use, could elucidate reasons for the observed ongoing HIV transmission among the UK MSM population. Studies have consistently found associations between increased sexual risk behavior, such as condomless anal sex and group sex [10-13], with recreational drug use, and longitudinal data has highlighted the bi-directional relationship between sexual pleasure and drug use [14]. Longitudinal data from Australia has demonstrated an association between drug use and increased risk of HIV infection, in particular the use of oral erectile dysfunction medication in combination with methamphetamines to enhance sexual pleasure [12], and similar evidence was recently reported from a US study that showed a clear link between increased sexual risk behavior and starting methamphetamine use [15]. Although not a new concept in the United States [16-18], a recent UK report on “chemsex” [19], which is defined as the use of certain sexually disinhibiting recreational drugs for facilitating or enhancing sex, has highlighted a need for more research into behaviors that put MSM at high risk of HIV and STI acquisition as a public health priority. Longitudinal data on recreational drug use, chemsex and associations with high risk sexual behaviors, such as group sex, in HIV negative or undiagnosed MSM would provide valuable insight into potential causes for the observed increases in HIV and STI acquisition among MSM in the United Kingdom.

Reducing the large proportion of MSM with undiagnosed HIV that potentially contribute to onward transmission of HIV is a public health priority [20], and data from HIV-negative or undiagnosed MSM in the United Kingdom are currently needed to inform and develop better provision of HIV testing options. Despite high coverage (86%) of HIV testing among MSM attending sexual health clinics [21], generally the frequency of HIV testing among UK MSM remains low (estimated 30% never tested, 75% not in past year) [4], and alternative ways to test for HIV, other than through sexual health clinics, are urgently required [1]. HIV self-testing (HIVST) was made legal in the United Kingdom in April 2015 [22] and is defined by the test being collected, performed, and interpreted in private by the individual who wants to know their HIV status [23]. HIVST has the potential to alleviate some of the perceived barriers to other forms of HIV testing, such as stigma, discrimination, and inaccessibility of health services [24], due to the environment the test is performed in, which may encourage more people to test. Increased HIV testing and resulting diagnoses could have prevention benefits if newly diagnosed men are more likely to use condoms and have fewer sexual partners after diagnosis [25,26]. However, it is not known whether the availability of HIVST will increase the diagnosis rates of HIV in the United Kingdom. The AURAH2 study will seek to collect information on the acceptability and uptake of HIVST as it becomes more widely available.

The expansion of HIV testing options is also of particular relevance since it was demonstrated that HIV transmission is preventable through ART in 2011 [27]. Evidence that ART greatly reduces onward sexual transmission of HIV in MSM [28], as well as heterosexuals [27,29], was demonstrated through the interim results of the PARTNER study [28]. Furthermore, the concept of treatment as prevention (TasP) has been widely explored as an HIV prevention strategy and is recommended in the British HIV Association’s treatment guidelines to prevent onward transmission [30]. However, access to and uptake of ART is dependent on a person knowing their HIV status and, in the United Kingdom, research has shown that although widespread ART coverage among MSM at a population level may reduce HIV infectivity, it is unlikely to reduce the number of HIV transmissions in the absence of increased coverage and frequency of HIV testing [31]. There is some evidence that sexual risk behavior declines after an HIV diagnosis, as it has been demonstrated that behavior is modified to prevent onward transmission [25,26]. However, this has not been explored in the context of TasP, and little is known regarding changes in sexual behavior during primary HIV infection (a period characterized by very high infectiousness) and on the variability in sexual risk behavior over time at an individual level (eg, the duration of periods of very high risk). There are cohort studies of MSM that provide some information on these issues from Europe, the United States, and Australia [32-35]; however, there have been no follow-up studies among individuals at risk of HIV infection in the United Kingdom, which the AURAH2 study will seek to address. The role of TasP is also critical in HIV-negative MSM’s sexual decision making and risk reduction behaviors and, as yet, is largely unexplored among UK HIV-negative MSM. Further investigation is needed, particularly in light of TasP, into the risk reduction strategies that HIV negative or undiagnosed MSM utilize at a community level [36], such as sero-sorting (choosing a partner of believed sero-concordant status), negotiated safety (condomless sex with a sero-concordant main partner), strategic positioning (choosing a different sexual position or practice depending on the sero-status of a partner), and withdrawal (in which the negative partner is receptive during intercourse but without ejaculation by his partner) [37]. To investigate the role of TasP in sexual decision making and risk reduction strategies, the AURAH2 study will collect data to inform on these themes, including information on knowledge of an HIV-positive partner’s viral load. Collection of longitudinal data will help describe the sexual behaviors and risk reduction strategies among HIV negative MSM and assess the extent to which patterns of sexual behavior and condomless sex change over time within individuals. This information will play a key role when developing effective, targeted HIV prevention strategies.

A further significant development for HIV prevention strategies that the AURAH2 study will provide information on is pre-exposure prophylaxis (PrEP), which has been used as an HIV prevention tool for HIV-negative men in the United States since 2012 [38]. Although PrEP is not currently available on the UK National Health Service (NHS), generic formulations have been increasingly available via websites. In 2015, the results from the UK PROUD study [39] and the French IPERGAY study [40] demonstrated that daily [39] and “on demand” [40] dosing of Truvada, the antiretroviral tablet used for PrEP, reduced the risk of HIV acquisition in HIV-negative men by 86%. There has been increasing community [41] and clinical [42] pressure to make PrEP available through the NHS. New PrEP websites that have been developed by activists [43,44] acknowledge the potential to access PrEP in a number of different ways that include ordering it online, which may challenge how the access and uptake of PrEP is monitored and may lead people to obtain PrEP without the appropriate counselling and follow-up [42]. Self-reported changes in attitudes, access to, and use of PrEP and factors associated with PrEP use by HIV negative men in the United Kingdom will be vital to inform policy and inform on acceptability and uptake of PrEP in sexually active HIV-negative gay men.

The landscape of HIV prevention is changing as concepts such as TasP and PrEP are introduced, and advances in HIV testing technologies potentially make testing for HIV more accessible. In conjunction with evolving HIV prevention strategies, emerging patterns in lifestyle choices that affect sexual behavior are important to consider if current and effective HIV prevention interventions are to be designed and implemented. The information provided by the AURAH2 study will contribute to the understanding of the social, psychological, and health-related factors that are linked to high-risk sexual behaviors that potentiate transmission of HIV. The study will provide data highly relevant to HIV prevention efforts among MSM and will help inform national policies aimed at reducing HIV incidence and increasing HIV testing in the United Kingdom.

### Study Aims and Objectives

The aim of the AURAH2 study is to evaluate the incidence and predictors of new infections among HIV-negative MSM at risk of acquiring HIV and to assess changes over time in risk behavior and testing practices within individuals.

The detailed study objectives are to assess:

1. In MSM without diagnosed HIV:

(i) the prevalence and correlates of specific sexual behaviors, including numbers of condomless sex partners, condomless sex with casual partners and partners of unknown HIV status, insertive/receptive condomless sex, and other specific higher-risk sexual activities such as group sex and chemsex

(ii) the number of condomless sex partners before, during, and after the estimated period of primary HIV-infection and time of HIV diagnosis in men who become infected during the study, as well as correlates of within-person changes in sexual behavior

(iii) the frequency and type of HIV testing accessed over time (sexual health clinic, self-testing, general practitioner, surgery, hospital, other)

2. The extent to which baseline demographic, socioeconomic, and health and lifestyle factors (including recreational drug use and chemsex) are predictive of subsequent levels of condomless sex, incident HIV infection, and HIV-testing behaviors

3. The association of attitudes to HIV transmission, disclosure, treatment, and prognosis, with high-risk sexual behaviors, HIV-testing behaviors, and subsequent HIV acquisition

4. The associations of participant characteristics, sexual behavior, and attitudes with reported use of, and willingness to consider use of, post exposure prophylaxis (PEP) and PrEP

## Methods and Design

### Study Design

AURAH2 is a prospective cohort study of UK MSM not diagnosed with HIV. Baseline information is collected on each participant through the AURAH study paper questionnaire [9], which is completed during a sexual health clinic attendance. Follow-up questionnaires are made available online every 4 months through the study website and consist of two brief and one extensive questionnaire per year. Online follow-up will continue for up to 3 years from the time a participant joined the study during the recruitment period in 2015.

### Population and Setting

HIV negative or undiagnosed MSM adults attending sexual health clinics at three sites in the United Kingdom for STI screening or HIV testing are eligible to take part in the study. The three clinical sites are as follows: The Mortimer Market Centre, London; 56 Dean Street Clinic, London; and the Claude Nicol Centre, Brighton.

These three clinical sites were chosen based on their ability to provide access to large numbers of MSM attending sexual health services and previous successful collaboration with the researchers for the AURAH study [9]. During the AURAH study recruitment process, the three sites demonstrated their ability to provide a broad sample of homosexually active men, including gay, bisexual, and non-gay identified MSM.

The eligibility criteria to join the study is (1) self-reported HIV-negative, (2) self-defining as MSM, (3) being aged 18 years or over, (4) attending or having previously attended for routine STI or HIV testing in the study clinics, and (5) willing to be contacted for longitudinal follow-up for up to a 3-year period.

### Sample Size

The sample size calculation was based on our objective to assess within-person changes in sexual behavior after receiving an HIV diagnosis. This outcome is more constrained by power than others because it relies on comparisons of participants within the group who are infected with HIV during follow-up. For sexual behavior classified as whether or not a man reports >3 condomless sex partners in the past 3 months, 85 new HIV diagnoses would be needed to detect, with 80% power and 5% significance level, the following changes: 17 (20%) men newly diagnosed switching from >3 to ≤3 condomless sex partners pre to post diagnosis, and 4 (5%) men newly diagnosed switching from ≤3 to >3 condomless sex partners pre to post diagnosis. With 1000 HIV-negative men initially enrolled in the study sample, assuming an annual HIV incidence of 4% (for high-risk MSM) and a dropout rate of 15% per year, 96 new HIV infections would be expected to accrue over a 3-year period. This sample size of 1000 should provide adequate power for the other objectives.

### Recruitment

Participants are recruited to the AURAH2 study through two separate recruitment routes. The recruitment route 1 group consists of HIV negative or undiagnosed MSM who were (1) enrolled in the AURAH cross-sectional study [9] from the three clinics detailed above during targeted recruitment of MSM (until March 2015) and (2) who had indicated interest in future follow-up on the AURAH study consent form. An email invitation to participate in the AURAH2 study was sent to this group from the AURAH2 study website in March 2015. Participants who joined the AURAH2 study from AURAH were assigned the same study number in their online follow-up as their original AURAH study number so that online follow-up could be linked to responses in the original cross-sectional study.

The recruitment route 2 group consists of HIV-negative or undiagnosed MSM who are prospectively recruited in person through the three clinic sites from March 2015 until December 2016. This group is directly consented into the AURAH2 study in their sexual health clinic and completes the baseline AURAH paper questionnaire during their clinic attendance. Online registration with the study website using a personal smartphone or iPad is explained during the consent procedure, or participants are contacted within 2 weeks with an email invitation to register.

### Consent

Consent for the study is gained through two mechanisms, according to the recruitment route. Participants from recruitment route 1 (contacted in March 2015) were required to complete an online consent form for the AURAH2 study. This was presented to them on the study website after they had read the online patient information sheet and prior to registration.

Consent for participants via recruitment route 2 is obtained at study enrollment in the clinic setting via a paper-based information sheet and consent form. Participants recruited through recruitment route 2 do not need to complete an additional online consent form as information on the AURAH2 study is provided in the Patient Information Sheet. In both consent processes, participants are (1) made aware of the study aims, (2) made aware that participation means they are expected to complete brief online questionnaires about sexual behavior and HIV testing on a regular basis over a 3-year period, (3) asked to provide their email address and mobile phone number and consent to receive reminders to complete the online questionnaires via email and/or text message, but are also told that there will be a maximum of two reminders by email followed by one text message if they do not respond, (4) asked to provide their full name and date of birth and made aware that this information will be used to link with matching data in UK national clinical databases including the national HIV/AIDS Reporting System (HARS) database (see clinical data), (5) made aware that results of any HIV test results from the day they joined the study, or that they self-report during the study period (up to 3 years), will be recorded and stored securely and separately from the study questionnaire, (6) made aware that they can withdraw from the study at any point and ask for their personal data to be deleted and that this will not affect their care at their GUM clinic, and (7) advised that should they wish to withdraw from the study they should send an email to a specified contact address to make this request.

### Online Registration Procedure

Participants are sent a maximum of three “invitation to register” messages via the study website (see below). The first contact is an email containing an individualized link, which, when selected, allows the recipient to register an account with the study website. A second similar reminder email is sent a week later to participants who have not registered, and finally a text message is sent a week after the second email (if a mobile phone number was provided during the consent procedure). In each email, participants are provided with information on how to opt out of the study and any further contact. Participants who do not register within 1 week after the two reminder emails and a text message have been sent are removed from the study lists and not contacted further.

### Website Design and Features

The AURAH2 website was designed to provide full information on the study to the general public and the study participants. The home page provides a login box that allows only registered participants to gain access to the study questionnaires by entering a username and password. Once a participant has registered and completed the first online questionnaire, automated reminder emails are sent every 4 months when follow-up questionnaires are due. Each reminder email informs the participant that a questionnaire is due for completion and contains a link to the study website homepage to login and access a questionnaire. The message also contains information on how to receive a username and password change prompt if login details have been forgotten. If a participant does not log in and complete a questionnaire, a second automated reminder email is sent 1 week after the initial email, and a final reminder is sent by text message a week later. If a participant does not log in and complete a questionnaire after the third reminder, no further contact is made until the next questionnaire is due, 4 months later.

The secure website provides facilities for content management including the ability to change information pages and add new items and details of study publications. The administration pages are accessible only to user accounts controlled by the study coordinator and data manager. From the administration pages of the website, the “invitation to register” and follow-up messages are managed and the status reports and questionnaire results data can be securely downloaded on a regular basis.

### Study Questionnaires

#### Baseline Data

Extensive baseline data are collected through the pen-and-paper AURAH baseline questionnaire, details of which have been published elsewhere [9]. The questionnaire gathers detailed information on demographics, socioeconomic factors, physical and psychological health and well-being, knowledge and understanding of HIV and antiretroviral treatment, lifestyle factors (smoking, alcohol, and recreational drug use), HIV testing, knowledge and use of PEP, and sexual behavior. Both recruitment routes use the baseline AURAH questionnaire for initial data collection, which takes approximately 20-25 minutes to complete.

#### Online Questionnaires

Online data collection every 4 months will be ongoing until 2018. It consists of a brief online questionnaire that assesses sexual risk behavior, HIV testing history, self-reported STIs, and use and frequency of chemsex drugs from the preceding 3 months. The basic 4-monthly online questionnaire takes approximately 5 minutes to complete. A more detailed questionnaire is undertaken on an annual basis that includes the information collected in the 4-month questionnaire and additional information on use of HIV testing preferences, PEP and PrEP, physical and psychological symptoms, and attitudes to HIV transmission. The annual online questionnaire takes approximately 20 minutes to complete.

**Figure 1:**
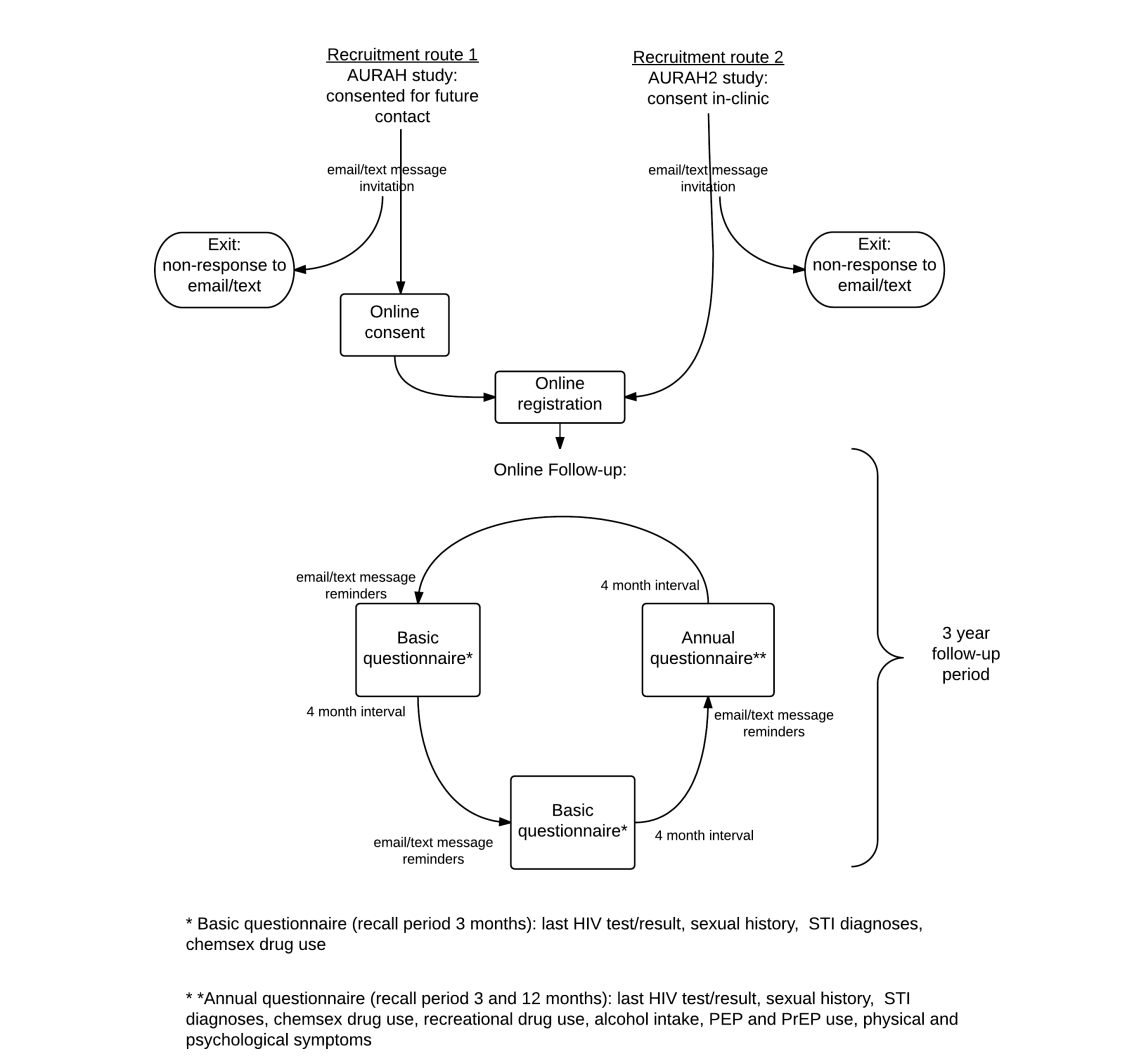
Flowchart of AURAH2 recruitment and questionnaire sequence.

Each online questionnaire commences with a question on the most recent date and result of a participant’s previous HIV test. If a participant consistently reports negative HIV test results or “not tested,” then the questionnaires remain specific to an HIV negative or unknown status. However, if a participant reports a positive HIV test result, the questionnaire is programmed to collect information on the number of partners, sexual behavior, and recreational drug use pre and post diagnosis. At each subsequent login, an HIV-positive participant will complete questionnaires that are similar to the HIV-negative participants, but tailored to reflect the HIV-positive sero-status of the participant. A flowchart to demonstrate recruitment from clinic to the online questionnaire sequence is shown in Figure 1.

### Clinical Data

The result of any HIV test taken in clinic at the same time as the baseline questionnaire completion is stored as part of the study records. At each online follow-up questionnaire, participants are asked to self-report the result of their most recent HIV test and any diagnosed STIs. At the end of the study period, in collaboration with Public Health England, data will be checked against corresponding records and data in national clinical databases such as HARS, the Genitourinary medicine clinical activity dataset (GUMCAD), and the Office for National Statistics. The linkage of the AURAH2 data to these databases will provide confirmation on the self-reported HIV status of participants as well as identify any new HIV diagnoses that have not been self-reported through the questionnaires.

### Data Processing and Security

Baseline paper questionnaires completed in clinic are collected by the study nurses and transferred regularly to the study management center via registered post or collected in person from the clinic sites by the study researchers. At the study management center, the original paper questionnaires are stored securely in locked cabinets. Baseline questionnaires are identifiable only by an assigned study number to maintain confidentiality, and participant details linked with the study number are collected in a separate study log. The study log is maintained securely and updated daily at each clinical site. The study log contains study numbers, clinic identifiers, and details of consent status for all patients invited to participate in the study, whether or not HIV and other STI tests had been done, and the result of any HIV test. Contact details of participants are also entered in the log. A copy of the study log (with contact details removed for non-consenting participants) is transferred on a regular basis to the study management center using the NHS mail system, which is approved by the Department of Health for the purpose of sharing personal identifiable information and sensitive information.

Baseline questionnaires are digitized at the management center using the REDCap data capture system for secure double data entry. The study website is hosted in a secure data center and network environment, and the online questionnaire response datasets are directly downloaded to encrypted data drives at the study management center on a monthly basis. Linkage to Public Health England’s datasets will be done at the end of the study using limited participant identifiers: surname Soundex, sex, and date of birth.

The final resulting study datasets, including scanned images of the questionnaires, are stored on the University College London Data Safe Haven, which is a secure technical solution for storing, handling, and analyzing identifiable data. This has been certified to the ISO27001 information security standard and conforms to the NHS Information Governance Toolkit.

The contact details of any participants who did not join the AURAH2 study after the email invitations from recruitment route 1 were removed from the study records 1 month after their final email reminder or the text message had been sent (if provided). All AURAH2 participants contact details will be erased from the study database 6 months after the completion of the study.

### Ethics Approval

The research protocol and all versions of the study documents (information sheet, consent form, questionnaires, and versions of the online questionnaires) were approved by the designated research ethics committee (NRES committee London-Hampstead, ref: 14/LO/1881 in November 2014). Based on these documents, the study subsequently received permission for clinical research at the three participating National Health Service sites: Chelsea and Westminster NHS Foundation Trust, Central and North West London NHS Foundation Trust, and the Brighton and Sussex University Hospitals NHS Trust.

## Results

Data collection commenced in March 2015 and is ongoing until March 2018. Initial results from analysis of the baseline questionnaires are expected in 2016, and results from longitudinal data are expected in 2018.

## Discussion

### Principal Considerations

The AURAH2 study will provide important longitudinal data on sexual risk behavior, HIV testing habits, and risk factors for ongoing transmission of HIV in UK MSM who were HIV negative at entry to the study but who are at risk of HIV infection. It uses a novel approach to data collection by combining paper-based questionnaires collected in the clinic setting and online follow-up questionnaires that participants access at their convenience. We applied a short recall period of 3 months in the questionnaire responses to maximize self-report accuracy and diminish recall bias [45] and to better capture within-person changes over time in sexual behavior. The request to complete questionnaires was sent on a 4-monthly basis to decrease the study burden for the participant and reduce attrition during the long follow-up period. The online recall period is reflective of the timeframes that are used in the paper-based questionnaires and is closely aligned to the frequency of survey completion in an attempt to capture ongoing and new behavioral information. The Internet has been increasingly used as a tool with which to collect survey data as it offers a low-cost, flexible, and fast way to collect data while reducing participant burden [46], and Internet surveys have been shown to be an acceptable method in researching MSM at risk of acquisition of HIV [47].

A similar study to AURAH2 is currently being conducted in the United States, using online follow-up over a 3-year period in a sample of 1000 gay and bisexual men who will complete self-administered HIV/STI tests and online surveys [48], which may offer comparisons of survey response rates and attrition over the 3 years. Despite some design differences, notably in recruitment routes (ie, the *One Thousand Strong* study recruited in partnership with a marketing firm via email invitation as opposed to face to face in sexual health clinics) and methods (ie, AURAH2 does not use biological methodologies for HIV/STI tests [48]), both studies will demonstrate the feasibility of using the Internet to engage MSM in online data collection and contribute substantial insight into sexual risk behavior and HIV testing. Longitudinal online follow-up in the HIV negative or undiagnosed population has not been widely explored among MSM in the United Kingdom but will be key to understanding how individual sexual behaviors change over time. The annual Gay Men’s Sex survey “Vital Statistics” has been successfully conducted among participants since 1993, although the survey is a one-off online survey [49], as opposed to the follow-up and retention of the same group of individuals over time. The AURAH2 study will provide valuable insight into the feasibility of recruiting and retaining MSM in a study that requires regular ongoing follow-up over a period of 3 years using the Internet as a tool for data collection.

### Limitations

A recognized limitation of the study is the restricted recruitment of MSM from attendance at GUM clinics, which may not be reflective of the wider MSM population, and in particular from the two clinics (56 Dean Street and the Mortimer Market Clinic) that provide services for patients seeking support for drug use. However, recruitment is from the general clinic attendees, not from specific drug use services. Although the majority of MSM in London do appear to be engaged with GUM sexual health services [50], there is less information on engagement with these services in the rest of the United Kingdom, so recruiting from a site outside London will allow some comparison. Recruitment through GUM clinics was essential for this study so that the self-reported HIV test results of participants could be confirmed with UK national clinical HIV databases (in collaboration with Public Health England) and so that the study sampling frame was clearly based on MSM who had attended a sexual health clinic. Recruitment online or through other settings could have potentially provided larger numbers of anonymous participants but would have been limited by the inability to confirm HIV status during or at the end of the study period due to participant anonymity.

We identify a further limitation of the study in the lack of recruitment from clinical sites outside two major cities that have large gay communities. It is recognized that this will limit the study’s generalizability given the potential differences in lifestyle, HIV testing opportunities, and access to sexual health services between urban and rural settings. Future studies might address this issue using the Internet or other digital platforms to enroll participants so that a broader sample of MSM from across the United Kingdom could be included.

### Conclusion

Evidence that HIV incidence is increasing among MSM in the United Kingdom [1] indicates a clear need for ongoing research in this group. The AURAH2 study will provide detailed longitudinal data on the incidence and predictors of new infections among HIV-negative MSM at particular risk of HIV infection and will further provide some of the first data on emerging behaviors such as chemsex that have raised concern for sexual health and well-being among MSM, as well as interest and uptake of PrEP and expanded HIV testing options for this group. The study completed recruitment of participants in March 2016, and it is hoped that the wide range of topics explored by the AURAH2 study renders results that will help improve a variety of targeted health promotion strategies that are specific to the men that need them. The study will be highly relevant to HIV prevention efforts among MSM, and it is planned for the data to feed into a mathematical model that simulates different scenarios to inform prevention strategies [4,51,52]. The results of the study will also inform national policies aimed at reducing HIV incidence and increase HIV testing in the United Kingdom in this population.

## Abbreviations

AIDS: acquired immune deficiency syndrome
ART: antiretroviral therapy
GUM: genito-urinary medicine
HIV: human immunodeficiency virus
HIVST: human immunodeficiency virus self-testing
MSM: men who have sex with men
NHS: National Health Service
Pep: post exposure prophylaxis
PrEP: pre-exposure prophylaxis
STI: sexually transmitted infection
TasP: treatment as prevention
